# Annotations

**Published:** 1903-05-02

**Authors:** 


### Annotations.

### Unregistered Foreign Practitioners.

The ease with which unregistered practitioners can obtain a practice in this country was demonstrated at an inquest held on April 24th on the body of Lieut.-Col. M. C. Garsia, C.B., one of his Majesty's Commissioners of Prisons, who died suddenly on April 20th at his private residence. The deceased gentleman consulted Dr. H. N. Dahkyl, M.D., B.Sc., B.A.Paris, of Holland Road, described as a specialist for deafness, ear, nose, and throat diseases, who prescribed for him. It appears that shortly after taking the medicine labelled "Compound Nutritive Syrup," Colonel Garsia complained of headache and a nasty taste in his mouth, and on the morning of his death wrote a letter to Dr. Dahkyl explaining the symptoms and stating that the medicine did not seem to agree with him. Soon after writing this letter alarming symptoms developed and Dr. Eric Pritchard was hastily summoned, and arrived shortly before death occurred. Owing to the fact that the deceased had been treated by an unregistered practitioner, to the suddenness of the death, and to the inconclusiveness of the symptoms, an inquest was ordered by the coroner. This case illustrates the very unsatisfactory condition of the law in relation to unregistered practitioners, which may cause those who employ them considerable inconvenience and unpleasantness. The public as a rule are not aware that death certificates are not accepted from unregistered practitioners, and consequently inquiry is seldom made, providing the practitioner adds to his name a number of letters signifying imposing degrees. As long as the patient recovers, the practitioner continues his career undisturbed, but should death ensue, both he and the patient's friends are placed in an awkward position, which is fair to neither. In Dr. Dahkyl's case the jury were satisfied that the medicine he prescribed had nothing to do with the cause of death. If from erroneous diagnosis he had administered a medicine injurious to the patient, he would not have had the protection enjoyed by the registered practitioner.

### Medical Missions.

The Medical Missions Auxiliary of the Church Missionary Society keeps up over 1,800 hospital beds abroad, distributed chiefly over India, Tropical Africa, and China. In charge of them are some seventy qualified medical practitioners whose function it is to spread the Gospel as well as the benefits of modern medical science. A great many of the practitioners thus employed are women, the society indeed apparently experiencing some difficulty in retaining the services of men. Much of the work done by Medical Missions, when their operations are confined to districts which ordinary Western civilisation has not reached is undoubtedly very good; and more support would probably be obtained for them if the reports in which they are recommended to the public were more judiciously worded. No one to whom the plain facts themselves do not appeal, is likely to be won over by the exaggerated expressions in which they are commonly clothed for publication. The sympathies, indeed, of many are probably turned aside by the adoption of an unctuous and emotional style which gives an appearance of cant and insincerity to work which in itself is solid and good. Another disfigurement is the frequent quotation of the speeches of native "converts" and patients. Those who are accustomed to dealing with the natives of any coloured race can translate for themselves the picturesque imagery with which they habitually express their thoughts and feelings. The ordinary English public, however, to whom these reports are addressed, having no such experience, either attaches much more importance to the speeches than they possess or deems them an unpleasant kind of "high faluting." Therefore, if native speeches are quoted at all, they should be accompanied by some such qualification as will bring them into proper focus. Such "editing" would lead to anything like constructive misrepresentation being avoided and make the reports more effective with those who are ready to support any good work but who are chilled by exaggeration.

### Death or Beauty.

The inquiry into the cause of death of a young girl who had managed to acquire perforation of the stomach through eating raw rice with a view to improving her complexion shows that the cult of beauty goes far beyond the region treated by Bond Street specialists. The lower down in the social scale the practice of fighting with unfriendly nature goes, the more unwise is it likely to become. The high-priests of Bond Street know something of the action of the drugs they recommend and the operations they employ, and as their clients have for the most part some notion of hygiene they are compelled to give the assurance that their methods are not dangerous to health. Wigs and a massaged complexion may not give the look of youth which those who adopt them imagine, but at least they do not shorten life, and the middle-aged woman who by their means seeks to keep up her pristine charm does so because life is still sweet. She may seek beauty, but she will not risk life for it. But the young girl—and especially the young girl of the humbler classes—who seeks to improve her appearance, does not consciously or unconsciously analyse the matter so. She wants to look pretty now, and hardly reckons on her future appearance. Below the middle classes matrimony remains the aim and end of feminine being. We all know how after marriage the smart and pretty girls who once outshone in beauty the maids of a higher station lapse into plain and untidy slatterns. The conquest of a lover once achieved, there is no need for further effort. To girls who look thus on life it is a matter of course that they should ape the style of beauty that is fashionable in their circle, regardless of its ultimate effect on their health. If John objects to a "red face" Mary will eat anything or drink anything to change her rosy complexion to a dyspeptic pallor. We know of one servant girl who was persuaded by her mistress to stop drinking vinegar to make herself pale by the assurance that rosy cheeks were now the fashion. After that Mary Jane daubed hers with rouge.

